# The correlation between the uric acid to high-density lipoprotein cholesterol ratio and stroke

**DOI:** 10.3389/fmed.2025.1720646

**Published:** 2026-01-13

**Authors:** Yuping Huang, Xiaoxiong Huang, Kun He, Weikun Zhang, Yuxi Liu, Luqiang Li, Lu Yuan, Fei Yang, Wen Zeng

**Affiliations:** 1 School of Public Health, Hengyang Medical School, University of South China, Hengyang, China; 2 Department of Public Health, The Central Hospital of Shaoyang, Shaoyang, China; 3 Department of Neurology and Stroke Center, The Central Hospital of Shaoyang, Hunan, China; 4 School of Public Health, Ningxia Medical University, Yinchuan, China; 5 The Affiliated Shaoyang Hospital, Hengyang Medical School, University of South China, Hengyang, China; 6 The School of Public Health, Guilin Medical University, Guilin, China

**Keywords:** stroke, uric acid to high-density lipoprotein cholesterol ratio (UHR), external validation, NHANES, restricted cubic spline (RCS), subgroup analysis

## Abstract

**Background:**

The uric acid to high-density lipoprotein cholesterol ratio (UHR) is emerging as a novel biomarker reflecting the balance between pro-oxidative and antioxidative pathways. While implicated in various cardiometabolic diseases, its specific correlation with stroke risk, particularly across diverse populations, remains insufficiently characterized. This study aimed to investigate the UHR-stroke link in two independent populations.

**Methods:**

This cross-sectional analysis utilized data from 27,439 NHANES participants (2007–2018). We employed survey-weighted multivariable logistic regression to model the UHR-stroke correlation, complemented by restricted cubic splines (RCS) for dose-response relationships and subgroup analyses to assess effect modification. Furthermore, an external validation dataset comprising 1780 patients from The Central Hospital of Shaoyang was recruited to independently validate the primary findings.

**Results:**

A significant positive correlation was consistently observed between elevated UHR levels and increased stroke risk in both populations. Each unit increase in UHR corresponded to an adjusted OR of 1.03 (95% CI: 1.01–1.05) in NHANES and 1.06 (95% CI: 1.02–1.10) in the Chinese dataset. Similarly, an increasing trend in risk was evident across higher UHR quartiles. Dose-response trends were evident in both datasets (*P* for trend < 0.05), with restricted cubic spline supporting a linear correlation. Subgroup analyses were robust across multiple sensitivity and multivariable-adjusted models.

**Conclusion:**

UHR is consistently and positively associated with stroke risk in both U.S. and Chinese populations. These findings, derived from multi-ethnic and external validation dataset, strengthen the evidence for UHR as a practical biomarker for stroke risk assessment, potentially reflecting underlying oxidative-antioxidant imbalance.

## 1 Introduction

Stroke stands as a leading cause of global mortality and permanent disability, imposing an immense burden on healthcare systems worldwide (1). Although significant strides have been made in acute ischemic stroke management—notably through endovascular thrombectomy and thrombolysis—a substantial burden of long-term disability persists (2, 3). For instance, despite improved early outcomes with reperfusion therapy, a considerable proportion of survivors still experience functional dependence months after the event (4–6). This underscores the critical need for better risk stratification and a deeper understanding of underlying pathophysiological mechanisms beyond traditional vascular risk factors like hypertension and diabetes.

One promising yet underexplored pathway is systemic oxidative stress, a key driver of endothelial dysfunction and atherosclerosis (7, 8). Uric acid (UA) and high-density lipoprotein cholesterol (HDL-C) are central, opposing players in this balance. UA, the end product of purine metabolism, acts as a pro-oxidant by stimulating the production of reactive oxygen species (ROS) and promoting inflammatory pathways, thereby contributing to endothelial dysfunction and atherosclerosis (9–11). In contrast, HDL-C exerts potent antioxidant and anti-inflammatory effects, primarily through its role in reverse cholesterol transport and by inhibiting the oxidation of low-density lipoprotein (12–15). However, epidemiological evidence on their individual links to stroke is complex and sometimes contradictory (13, 16–22), likely confounded by metabolic syndrome components and their interdependent biological roles. This complexity limits the predictive utility of measuring UA or HDL-C alone (13, 20–23).

The Uric acid to high-density lipoprotein cholesterol ratio (UHR) may overcome this limitation by integrating both facets into a single metric, theoretically reflecting the net balance between pro-oxidative and antioxidative capacities. This integrative approach is biologically plausible because the pathogenic impact of high UA might be modulated by concurrent antioxidant levels of HDL-C. Consequently, UHR has demonstrated superior predictive value for several cardiometabolic conditions compared to its individual components (24–26).

Despite this promise, direct evidence linking UHR to stroke risk remains scarce. A recent cross-sectional study reported a positive correlation (27), but evidence from a single population is insufficient. There is a clear need for external validation in independent cohorts and more detailed analyses of key subgroup consistency. Thus, whether the UHR-stroke correlation is generalizable beyond single populations, and robust across demographic and clinical strata remains unclear.

Therefore, we conducted this comprehensive study with three primary aims: (1) to investigate the independent correlation between UHR and stroke prevalence in a large, multi-ethnic U.S. representative sample (NHANES) and to validate this finding in an independent Chinese clinical datasets; (2) to assess the consistency of the UHR-stroke correlation across key demographic and clinical subgroups; and (3) to specifically evaluate this correlation stratified by sex, and to descriptively compare it with the sex-stratified correlation between UA alone and stroke. By addressing these gaps, our study aims to provide robust, multidimensional evidence supporting UHR as a practical biomarker for stroke risk, rooted in the pathophysiology of oxidative-antioxidant imbalance.

## 2 Materials and methods

### 2.1 Study design and data source

The U.S. National Health and Nutrition Examination Survey (NHANES) is a nationally representative program assessing the health and nutritional status of the non-institutionalized U.S. population (28). Conducted in biannual cycles, NHANES collects data through standardized household interviews and physical examinations at mobile examination centers (MECs). The survey employs a multistage stratified probability sampling design to ensure population representativeness. This cross-sectional study utilized NHANES data from six survey cycles (2007–2018). The protocol was approved by the National Center for Health Statistics (NCHS) Research Ethics Review Board, and written informed consent was obtained from all participants by the Centers for Disease Control and Prevention (CDC). Full ethics documentation is available at: https://www.cdc.gov/nchs/nhanes/about/erb.html.

For external validation, we conducted a retrospective study using clinical data obtained from inpatient and outpatient records at The Central Hospital of Shaoyang between January 2024 and June 2025. The study was approved by the institutional ethics committee. As only anonymized retrospective data were used without compromising privacy or imposing additional risks, informed consent was waived following applicable guidelines.

### 2.2 Definition of stroke

Stroke status was ascertained using self-reported data from the Medical Conditions Questionnaire (MCQ) (29). Participants were classified as having stroke if they answered “yes” to the question: “Has a doctor or other health professional ever told you that you had a stroke?” This standardized instrument was administered by trained interviewers and has been validated in prior studies examining stroke prevalence (30–32). Although NHANES does not provide stroke subtype classification, population-based studies suggest that a majority of stroke survivors in the US have ischemic etiology (33). Our analysis, therefore, primarily reflects correlations with ischemic stroke, which is more closely linked to chronic inflammatory pathways (34–37). Notably, we acknowledge a key limitation: potential recall bias in self-reported diagnoses, particularly among elderly individuals with cognitive impairment.

In the Chinese dataset, stroke cases were defined as patients with a clinical diagnosis of stroke, made by a neurologist based on acute neurological deficits and confirmed by cranial computed tomography (CT) or magnetic resonance imaging (MRI). All diagnoses were documented in the hospital’s electronic medical record system.

### 2.3 Assessment of UHR

UA and HDL-C levels were obtained from NHANES physical examinations (2007–2018). Fasting blood samples were collected by venipuncture following standardized protocols (38). HDL-C was quantified using a direct enzymatic method (39): A magnesium sulfate/dextran solution was added to the sample, forming water-soluble complexes with non-HDL cholesterol, which did not react with the measurement reagents in subsequent steps. Then, by adding polyethylene glycol esterase, HDL-C esters were converted to HDL-C. The hydrogen peroxide generated in this reaction reacted with 4-aminoantipyrine and HSDA to form a purple or blue dye. Finally, laboratory researchers determined HDL-C levels by photometric measurement at 600 nm. The steps for UA measurement were as follows (39): UA concentration was measured using the timed endpoint method with a DxC800 automated chemical analyzer. UA was oxidized by uricase to produce allantoin and hydrogen peroxide. Hydrogen peroxide reacted with 4-aminoantipyrine (4-AAP) and 3,5-dichloro-2-hydroxybenzenesulfonate (DCHBS) in a peroxidase-catalyzed reaction to produce a colored product, which was then measured photometrically at 520 nm to determine UA levels. In this study, the UHR was calculated using the following formula:


[UA⁢(mg/dL)/HDL-C⁢(mg/dL)]×100⁢(40,41).

In the Chinese dataset, UA and HDL-C were measured from fasting blood samples using standard automated biochemical analyzers, and UHR was calculated identically.

### 2.4 Covariates

Demographic data were collected via standardized questionnaires and in-person interviews. The following potential confounders were adjusted for in the analyses: age, sex, race (Mexican American, Other Hispanic, Non-Hispanic White, Non-Hispanic Black, Other), marital status (formerly married, married or never married), and education level (below high school, high school, above high school). BMI was calculated as weight divided by height squared (kg/m^2^) and categorized as < 25, 25–29.9, or ≥ 30 kg/m^2^. Lifestyle factors included smoking and drinking status. Smoking status was categorized based on the question “Do you now smoke cigarettes?”. Drinking status was defined as a binary variable, classifying participants who reported having at least 12 drinks in the past year as “drinkers” and others as “non-drinkers” (42). Hypertension was defined as systolic blood pressure ≥ 140 mmHg, diastolic blood pressure ≥ 90 mmHg, self-reported diagnosis, or current use of antihypertensive medication (43). Diabetes was defined based on self-reported diagnosis, use of glucose-lowering medication, fasting glucose ≥ 126 mg/dL, or HbA1c ≥ 6.5% (44). Coronary heart disease (CHD) was identified through self-reported physician diagnosis of coronary heart disease, angina, or heart attack (45). Detailed variable definitions are available at: https://wwwn.cdc.gov/nchs/nhanes/. Covariates from the Chinese dataset included age, sex, careers, SBP, DBP, BMI, lifestyle factors (smoking, drinking), history of Hypertension, Diabetes, Liver disease, Kidney disease, Tumor, and laboratory measures.

### 2.5 Statistical analysis

All analyses incorporated NHANES complex sampling weights per NCHS guidelines to ensure national representativeness. We applied the recommended mobile examination center (MEC) sample weights (WTMEC2YR). Analyses also accounted for primary sampling units (SDMVPSU) and strata (SDMVSTRA) using the survey package in R to produce nationally representative estimates. Continuous variables are presented as weighted mean ± standard deviation or median (interquartile range), and categorical variables as unweighted counts (weighted percentages). Group comparisons utilized weighted *t*-tests or ANOVA for normal variables, and weighted Mann-Whitney U or Kruskal-Wallis tests for non-normal variables, with normality assessed using weighted Kolmogorov-Smirnov tests.

The correlation between UHR (as a continuous variable and in quartiles) and stroke was assessed using multivariable logistic regression. We built three sequential models: Model 1 (Crude) included UHR only; Model 2 adjusted for demographics (age, sex, race); Model 3 (Fully Adjusted) additionally adjusted for BMI, smoking, drinking, hypertension, diabetes, and CHD. Variance inflation factors (VIF) were examined to assess multicollinearity (all VIF < 5). To examine the dose-response relationship, we fitted restricted cubic splines (RCS) with five knots placed at the 5th, 27.5th, 50th, 72.5th, and 95th percentiles of the UHR distribution. The linearity of the correlation was tested by comparing the model with the spline terms against a linear-only model using a likelihood ratio test. Subgroup analyses were performed by fitting the fully adjusted model within strata defined by sex, age, etc. Interaction was tested by including a product term between UHR and the subgroup variable. A two-sided *P*-value < 0.05 was considered significant. All analyses were performed using R software (version 4.4.0).

## 3 Results

**FIGURE 1:**
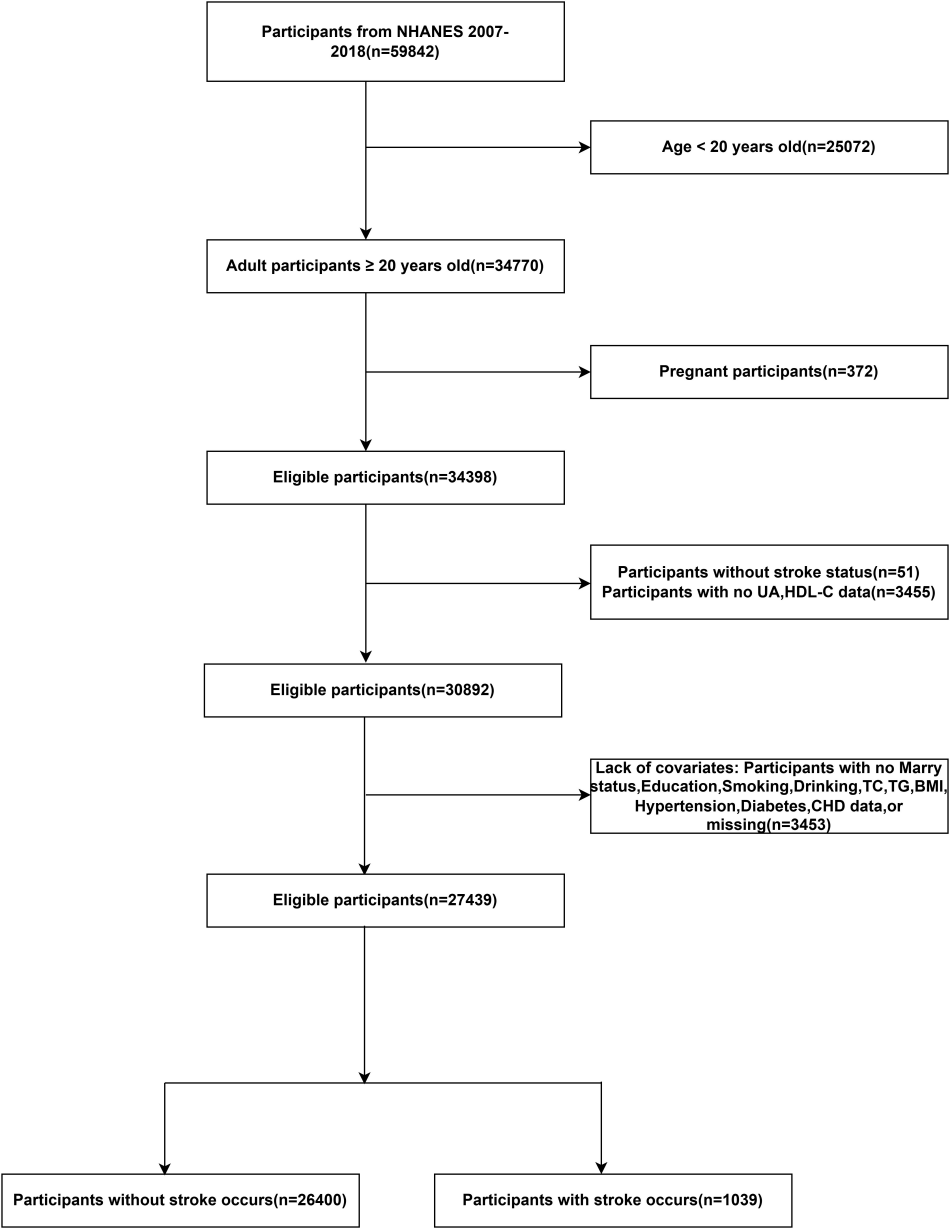
Participant selection flowchart for the NHANES.

A total of 59,842 participants from six consecutive NHANES cycles (2007–2018) were initially considered. After excluding individuals aged < 20 years, pregnant participants and those with missing data for stroke status, UA, HDL-C, or any of the key covariates (age, sex, smoking, drinking, hypertension, diabetes, coronary heart disease), 27,439 adults were included in the final analysis. Missing data were handled using a complete-case analysis approach. The detailed selection process is illustrated in Figure 1.

Inclusion criteria for the Chinese dataset were: (1) age ≥ 20 years; (2) clinically confirmed stroke with neuroimaging support; (3) available UA and HDL-C measurements. Exclusions included missing key data, repeated admissions (only first record kept), malignancy, pregnancy, or severe organ failure. To handle missing data, multiple imputation by chained equations (MICE) was employed to mitigate potential bias. *Post hoc* completion, outlier detection, and exclusion were performed to ensure data integrity (46). 1,429 participants constituted the final validation sample.

### 3.1 Baseline characteristics of the study participants

Table 1 summarizes the baseline characteristics of the NHANES participants stratified by stroke status. The final analytical sample included 27,439 individuals (weighted *N* = 191,884,477), of whom 1,039 (weighted *N* = 5,432,504) had a self-reported stroke. Participants with stroke were generally older and had a higher prevalence of smoking, lower educational attainment, and a greater comorbidity burden. Metabolically, they exhibited significantly elevated median UHR (11.4 vs. 10.5; *P* < 0.001), higher triglycerides (134 vs. 121 mg/dL; *P* < 0.001), and lower HDL-C levels (48 vs. 51 mg/dL; *P* < 0.001).

**TABLE 1:** Baseline characteristics of the NHANES population stratified by stroke.

Characteristic	N^1^	Overall *N* = 191,884,477^2^	Non-stroke *N* = 186,451,974^2^	Stroke *N* = 5,432,504^2^	*p*-value^3^
Sex, n (%)	27,439				0.022
Male		13,668 (49%)	13,156 (50%)	512 (45%)	
Female		13,771 (51%)	13,244 (50%)	527 (55%)	
Age, n (%)	27,439				< 0.001
20–30		4,800 (20%)	4,783 (20%)	17 (2.2%)	
31–40		4,457 (17%)	4,427 (17%)	30 (3.4%)	
41–50		4,576 (19%)	4,484 (19%)	92 (11%)	
51–60		4,705 (19%)	4,531 (19%)	174 (19%)	
>60		8,901 (25%)	8,175 (24%)	726 (65%)	
Race, n (%)	27,439				< 0.001
Mexican American		4,167 (8.4%)	4,077 (8.6%)	90 (4.4%)	
Other Hispanic		2,876 (5.7%)	2,812 (5.8%)	64 (2.7%)	
Non-Hispanic White		11,660 (68%)	11,137 (68%)	523 (71%)	
Non-Hispanic black		5,621 (10%)	5,332 (10%)	289 (15%)	
Other race		3,115 (7.5%)	3,042 (7.5%)	73 (7.6%)	
Marital status, n (%)	27,439				< 0.001
Formerly married		6,146 (18%)	5,746 (18%)	400 (34%)	
Married		16,314 (63%)	15,768 (64%)	546 (58%)	
Never married		4,979 (18%)	4,886 (18%)	93 (7.7%)	
Education level, n (%)	27,439				< 0.001
Below high school		6,520 (15%)	6,169 (15%)	351 (26%)	
High School		6,299 (23%)	6,009 (23%)	290 (31%)	
Above high school		14,620 (62%)	14,222 (62%)	398 (43%)	
BMI, n (%)	27,439				0.010
<25		7,694 (29%)	7,441 (29%)	253 (25%)	
25–29.9		9,052 (33%)	8,726 (33%)	326 (31%)	
≥30		10,693 (38%)	10,233 (38%)	460 (44%)	
Smoking, n (%)	27,439				< 0.001
No		15,136 (55%)	14,742 (56%)	394 (40%)	
Yes		12,303 (45%)	11,658 (44%)	645 (60%)	
Drinking, n (%)	27,439				< 0.001
No		6,900 (20%)	6,602 (20%)	298 (26%)	
Yes		20,539 (80%)	19,798 (80%)	741 (74%)	
TC (mg/dL)	27,439	190 (164, 218)	191 (165, 218)	178 (153, 210)	< 0.001
HDL-C (mg/dL)	27,439	51 (42, 62)	51 (42, 62)	48 (40, 61)	< 0.001
TG (mg/dL)	27,439	121 (81, 186)	121 (80, 186)	134 (92, 197)	< 0.001
UA (mg/dL)	27,439	5.4 (4.4, 6.3)	5.4 (4.4, 6.3)	5.6 (4.6, 6.8)	< 0.001
UA (male)	13,668	6.0 (5.2, 6.8)	6.0 (5.2, 6.8)	6.0 (5.1, 7.1)	0.809
UA (female)	13,771	4.7 (4.0, 5.5)	4.7 (4.0, 5.5)	5.2 (4.2, 6.5)	< 0.001
UHR	27,439	10.6(7.6, 14.3)	10.5 (7.5, 14.2)	11.4 (8.4, 15.3)	< 0.001
UHR (male)	13,668	13.0 (10.2, 16.6)	13.0 (10.2, 16.6)	14.0 (10.8, 17.4)	0.005
UHR (female)	13,771	8.3 (6.3, 11.0)	8.2 (6.2, 11.0)	9.4 (7.1, 12.9)	< 0.001
Diabetes, n (%)	27,439				< 0.001
No		22,348 (86%)	21,721 (87%)	627 (64%)	
Yes		5,091 (14%)	4,679 (13%)	412 (36%)	
Hypertension, n (%)	27,439				< 0.001
No		15,379 (61%)	15,181 (62%)	198 (22%)	
Yes		12,060 (39%)	11,219 (38%)	841 (78%)	
CHD, n (%)	27,439				< 0.001
No		25,398 (94%)	24,701 (95%)	697 (67%)	
Yes		2,041 (6.1%)	1,699 (5.3%)	342 (33%)	

Normally distributed values in the table are given as the mean ± SD, skewed distributed values are given as the median (25 and 75% interquartiles), and categorical variables are given as frequency (percentage). UHR uric acid to high-density lipoprotein cholesterol ratio, BMI Body mass index, UA uric acid, TG Triglyceride, TC Total cholesterol, HDL-C High-density lipoprotein cholesterol, CHD coronary heart disease.

To externally validate these findings, we established an independent clinical dataset comprising 1,429 participants from The Central Hospital of Shaoyang (Table 2). This dataset included 547 patients with imaging-confirmed stroke and 882 non-stroke controls. Consistent with the NHANES findings, stroke patients in the Chinese datasets were significantly older (67 vs. 61 years, *P* < 0.001) and demonstrated a more adverse metabolic profile, including higher UHR (12.88 vs. 10.83, *P* < 0.001), lower HDL-C (44.08 vs. 47.56 mg/dL, *P* < 0.001), and higher systolic and diastolic blood pressure (all *P* < 0.001). They also had a significantly higher prevalence of hypertension (76% vs. 37.0%, *P* < 0.001) and diabetes (31% vs. 20%, *P* < 0.001). No significant differences were found in the history of kidney disease, CHD, or tumors between the groups.

**TABLE 2:** Participants demographics and baseline characteristics in Shaoyang area.

Characteristic	*N*	Overall *N* = 1,429^1^	Non-stroke *N* = 882^1^	Stroke *N* = 547^1^	*p*-value^2^
Sex, n (%)	1,429				< 0.001
Male		792 (55%)	414 (47%)	378 (69%)	
Female		637 (45%)	468 (53%)	169 (31%)	
Age	1,429	62.00(54.00,73.00)	61.00(51.00,72.00)	67.00(58.00,74.00)	< 0.001
BMI	1,429	23.08(20.76, 25.39)	22.89(20.55,25.15)	23.50(20.76,25.83)	0.008
Smoking	1,429				< 0.001
No		1,269 (89%)	812 (92%)	457 (84%)	
Yes		160 (11%)	70 (7.9%)	90 (16%)	
Drinking	1,429				< 0.001
No		1,335 (93%)	842 (95%)	493 (90%)	
Yes		94 (6.6%)	40 (4.5%)	54 (9.9%)	
Careers	1,429				0.048
Farmers		819 (57%)	487 (55%)	332 (61%)	
Workers		610 (43%)	395 (45%)	215 (39%)	
Hepertension	1,429				< 0.001
No		689 (48%)	556 (63%)	133 (24%)	
Yes		740 (52%)	326 (37%)	414 (76%)	
Diabetes	1,429				< 0.001
No		1,085 (76%)	708 (80%)	377 (69%)	
Yes		344 (24%)	174 (20%)	170 (31%)	
CHD	1,429				0.249
No		1,080 (76%)	657 (74%)	423 (77%)	
Yes		349 (24%)	225 (26%)	124 (23%)	
Liver disease	1,429				< 0.001
No		1,329 (93%)	802 (91%)	527 (96%)	
Yes		100 (7.0%)	80 (9.1%)	20 (3.7%)	
Kindey disease	1,429				0.604
No		1,327 (93%)	822 (93%)	505 (92%)	
Yes		102 (7.1%)	60 (6.8%)	42 (7.7%)	
Tumor	1,429				0.195
No		1,371 (96%)	841 (95%)	530 (97%)	
Yes		58 (4.1%)	41 (4.6%)	17 (3.1%)	
SBP	1,429	136.00 (124.00,150.00)	131.00 (122.00,143.00)	145.00 (134.00,158.00)	< 0.001
DBP	1,429	80.00 (72.00, 87.00)	78.00 (71.00, 85.00)	82.00 (75.00, 90.00)	< 0.001
UA (mg/dL)	1,429	5.34 (4.44, 6.36)	5.17 (4.29, 6.17)	5.62 (4.79, 6.80)	< 0.001
UA (male)	792	5.77 (4.92, 6.91)	5.74 (4.81, 6.73)	5.82 (5.00, 7.06)	0.057
UA (female)	637	4.85 (4.10, 5.76)	4.77 (3.99, 5.58)	5.15 (4.44, 6.05)	< 0.001
UHR	1,429	11.62 (8.93, 15.02)	10.83 (8.19, 14.18)	12.88 (9.84, 15.96)	< 0.001
UHR (male)	792	13.44 (10.45, 16.37)	13.18 (10.12, 16.17)	13.71 (10.74, 16.84)	0.016
UHR (female)	637	9.73 (7.47, 12.47)	9.42 (7.04, 11.91)	10.58 (8.81, 13.73)	< 0.001
TC (mg/dL)	1,429	173.24 (144.24,201.08)	174.79 (147.72,203.79)	167.05 (136.12,199.54)	0.003
HDL-C (mg/dL)	1,429	46.02(39.06, 54.91)	47.56(39.83, 56.84)	44.08(37.51, 51.43)	< 0.001
TG (mg/dL)	1,429	115.14(77.06, 168.28)	113.37(75.28, 165.63)	117.80(79.71, 173.60)	0.182
LDL-C (mg/dL)	1,429	100.54(78.11, 122.58)	100.74(79.27, 122.58)	100.54(77.34, 123.36)	0.694

Normally distributed values in the table are given as the mean ± SD, skewed distributed values are given as the median (25 and 75% interquartiles), and categorical variables are given as frequency (percentage) UHR uric acid to high-density lipoprotein cholesterol ratio, BMI, Body mass index; UA, uric acid; TG, Triglyceride; TC, Total cholesterol; LDL-C, Low-density lipoprotein cholesterol; HDL-C, High-density lipoprotein cholesterol; SBP, Systolic Blood Pressure; DBP, Diastolic Blood Pressure; CHD, coronary heart disease.

### 3.2 Correlation of UHR with stroke

Table 3 presents the correlation between UHR and stroke risk in the NHANES dataset. Multivariable weighted logistic regression revealed a consistent positive correlation across all models. Each unit increase in UHR (continuous) was associated with significantly elevated stroke odds in both the crude (Model 1: OR = 1.04, 95% CI: 1.02–1.05; *P* < 0.001) and fully adjusted models (Model 3: OR = 1.03, 95% CI: 1.01–1.05; *P* = 0.006). Similarly, a significant dose-response relationship was observed when UHR was analyzed in quartiles (*P* for trend < 0.05 for all models). Participants in the highest UHR quartile had a 44% increased risk of stroke compared to the lowest quartile (Model 3: OR = 1.44, 95% CI: 1.09–1.91; *P* = 0.012). The attenuation of the effect size from Model 1 to Model 3 suggests that cardiometabolic factors may partially mediate this correlation.

**TABLE 3:** The correlation between UHR and stroke. (NHANES)

Character	Model 1		Model 2		Model 3	
	OR (95% CI)	*P*-value	OR (95% CI)	*P*-value	OR (95% CI)	*P*-value
UHR	1.04 (1.02, 1.05)	< 0.001	1.05 (1.04, 1.07)	< 0.001	1.03 (1.01, 1.05)	0.006
**UHR (quartile)**						
Q1	Reference	Reference	Reference	Reference	Reference	Reference
Q2	1.15(0.87, 1.52)	0.300	1.24(0.93, 1.65)	0.140	1.13(0.84, 1.51)	0.400
Q3	1.19(0.92, 1.53)	0.200	1.34(1.03, 1.73)	0.028	1.10(0.84, 1.45)	0.500
Q4	1.63(1.29, 2.07)	< 0.001	2.05(1.59, 2.65)	< 0.001	1.44(1.09, 1.91)	0.012
*P* for trend		< 0.001		<0.001		0.026

CI, Confidence Interval; OR, Odds Ratio. Model 1: no covariates were adjusted. Model 2: Age, Sex and Race were adjusted. Model 3: Age, Sex, Race, BMI, Smoking, Drinking, Diabetes, Hypertension, and CHD were adjusted. UHR is categorized into quartiles (Q1–Q4).

To independently validate these findings, we performed identical analyses in the Chinese dataset (Table 4). Consistent with the NHANES results, a higher UHR was significantly associated with increased stroke risk. Each unit increase in UHR was associated with a 6% elevated risk in the fully adjusted model (OR = 1.06, 95% CI: 1.02–1.10; *P* = 0.005). When analyzed in quartiles, participants in the highest quartile (Q4) had 59% higher odds of stroke compared to those in the lowest quartile (Q1) (OR = 1.59, 95% CI: 0.98–2.57; *P* = 0.060), although this result was of borderline significance. A consistent dose-response trend was observed across quartiles in the minimally adjusted models (*P* for trend < 0.001), which, although attenuated, persisted in the fully adjusted model (*P* for trend = 0.084).

**TABLE 4:** Correlation between UHR and stroke in Shaoyang area.

Characteristic	Model 1			Model 2			Model 3		
	OR	95% CI	*p*-value	OR	95% CI	*p*-value	OR	95% CI	*p*-value
UHR (continuous)	1.11	1.08, 1.13	< 0.001	1.07	1.04, 1.11	< 0.001	1.06	1.02, 1.10	0.005
**UHR**									
Q1	–	–		–	–		–	–	
Q2	2.28	1.64, 3.17	< 0.001	1.87	1.29, 2.72	< 0.001	1.51	1.01, 2.24	0.043
Q3	2.78	2.00, 3.85	< 0.001	2.24	1.54, 3.27	< 0.001	1.74	1.13, 2.68	0.012
Q4	3.38	2.44, 4.68	< 0.001	2.20	1.49, 3.25	< 0.001	1.59	0.98, 2.57	0.060
*P* for trend			< 0.001			<0.001			0.084

CI, Confidence Interval; OR, Odds Ratio. Model 1: no covariates were adjusted. Model 2: adjusted for Age, Sex, Careers, Smoking, Drinking, Hepertension, Diabetes, CHD, Liver disease, Kindey disease, and Tumor. Model 3: adjusted for Age, Sex, Careers, Smoking, Drinking, Hepertension, Diabetes, CHD, Liver disease, Kindey disease, Tumor, SBP, DBP, BMI, TC, TG, HDL-C, and LDL-C.

### 3.3 Dose-response relationship (restricted cubic spline analysis)

**FIGURE 2:**
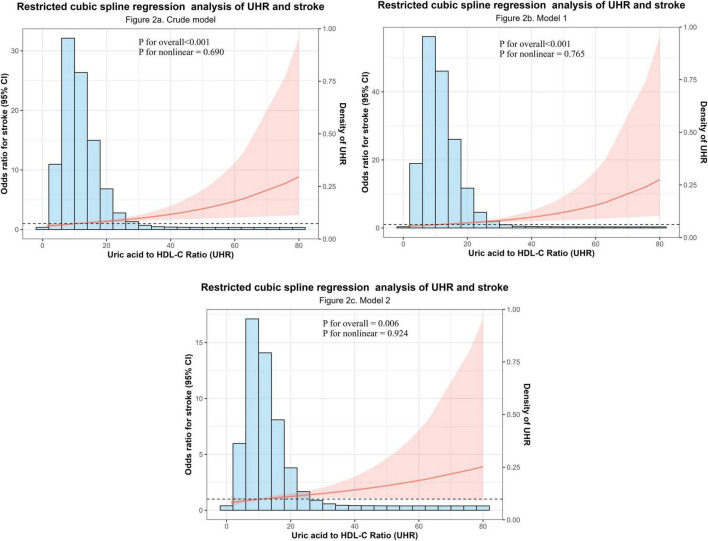
Dose-response relationship between the Uric acid to high-density lipoprotein cholesterol ratio (UHR) and stroke odds. **(a)** Crude model (unadjusted). **(b)** Partially adjusted model (adjusted for sex, age, and race). **(c)** Fully adjusted model (additionally adjusted for BMI, smoking, drinking, hypertension, diabetes, and coronary heart disease). *P*-values for the overall correlation and the non-linearity test are displayed within each panel.

To further investigate the correlation between UHR and stroke in the NHANES dataset, the RCS curve was used to illustrate the dose-response relationship of UHR and the likelihood of developing stroke (Figure 2). In the overall population, restricted cubic spline analysis revealed significant overall correlations between UHR and stroke in both unadjusted (Figure 2a) (*P* overall < 0.001) and partially adjusted models (Figure 2b) (*P* overall < 0.001), with no evidence of non-linearity (*P* non-linear = 0.690 for Figure 2a and 0.765 for Figure 2b) (Figure 2). This relationship persisted in the fully adjusted model (Figure 2c) with combined adjustment for demographics, lifestyle, and comorbidities (*P* overall = 0.006, *P* non-linear = 0.924). The dose-response curve demonstrated a monotonically increasing trend, indicating a consistent rise in stroke prevalence with higher UHR levels across its distribution. Critically, this linear relationship remained robust after multivariable adjustment, reinforcing UHR as a continuous risk marker for stroke.

### 3.4 Results of subgroup analyses

**FIGURE 3:**
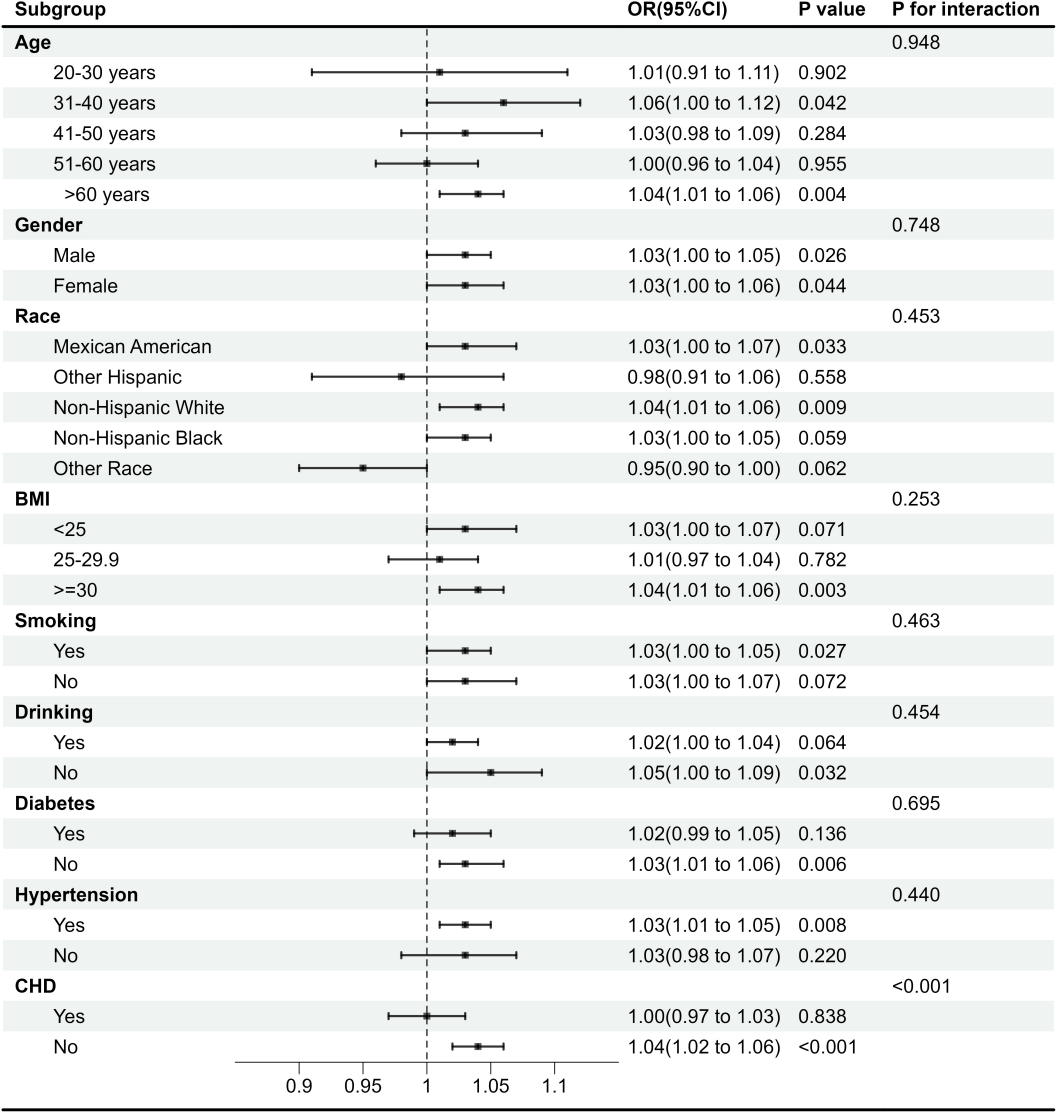
Subgroup analysis of the correlation between UHR and stroke risk.

Subgroup analyses stratified by sex, age, race, BMI, smoking, drinking, diabetes, hypertension, and CHD evaluated the robustness of the UHR-stroke correlation and identified potential effect modifiers (Figure 3). The positive correlation between UHR and stroke remained consistent across most demographic and clinical subgroups, including sex (male/female), specific age groups (31–40 years and > 60 years), key racial categories (Mexican American and Non-Hispanic White), BMI strata (≥ 30 kg/m^2^), smoking (smokers), drinking (no drinkers) and comorbidity profiles (non-diabetic, hypertensive, and non-CHD groups), with all showing statistically significant correlations (*P* < 0.05) (Figure 3). Significant effect modification by CHD status was observed (*P* for interaction < 0.001), where each unit increase in UHR conferred a 4% higher stroke risk in the CHD subgroup (OR = 1.04, 95% CI: 1.02–1.06), indicating a substantially amplified effect in individuals with established CHD. This enhanced correlation in the CHD population identifies patients with concomitant elevated UHR as a distinct high-risk phenotype, suggesting that targeted management of UHR may provide effective stroke risk mitigation in this vulnerable group through optimized metabolic control. Collectively, UHR demonstrates robustness as a stroke risk marker across diverse populations, with CHD status significantly modifying its predictive effect. The stronger correlation observed in the cardiovascular population underscores the need for early identification and management of metabolic and cardiovascular risk in this population to mitigate stroke risk effectively.

## 4 Discussion

Our study provides robust, two independent populations evidence that a higher UHR is independently associated with an increased prevalence of stroke. This positive correlation was consistent across both a nationally representative U.S. sample (NHANES) and an independent Chinese clinical population, whether UHR was analyzed as a continuous or categorical (quartile) variable. Key findings include a 3% increase in stroke odds per unit rise in UHR in the fully adjusted NHANES model, a 44% higher risk in the top versus bottom UHR quartile, and a linear dose-response relationship confirmed by restricted cubic spline analysis.

Our findings, which demonstrate a consistent and positive correlation between elevated UHR and increased stroke risk across both the NHANES and the independent Chinese clinical population, are in line with the recent report by Zhu et al., in a single population (27). While confirming the core correlation, our study provides critical advancements in three areas: generalizability, through validation in two ethnically and geographically distinct populations; depth of analysis, through comprehensive sex-stratified evaluations, specifically, we reported the sex-stratified median (IQR) of uric acid at baseline and assessed the UHR-stroke correlation via logistic regression analyses conducted separately in men and women (detailed in Supplementary Tables 1–4); and robustness, as demonstrated by consistent results across extensive subgroup analyses adjusting for key confounders including smoking and drinking status. Notably, the correlation appeared stronger in adults over 60 years and in individuals without coronary heart disease, highlighting populations where UHR may have particular clinical relevance. Mechanistically, elevated UHR in older adults reflects synergistic oxidative damage from age-related declines in renal UA clearance and HDL-C antioxidant capacity, potentially accelerating endothelial dysfunction and plaque instability. UHR can be used as a specific indicator of stroke risk in the elderly and be prioritized for use in geriatric physical examination programs. In CHD-free individuals, elevated UHR serves as an early metabolic risk marker warranting intensified lifestyle interventions (e.g., Mediterranean diet) prior to pharmacological management.

The biological plausibility of our findings is grounded in the role of UHR as an integrative marker of systemic oxidative-antioxidant imbalance. UA, the terminal product of purine metabolism, acts primarily as a pro-oxidant at high concentrations, stimulating reactive oxygen species (ROS) generation, promoting endothelial inflammation and dysfunction (19, 47–50), and is linked to insulin resistance. Conversely, HDL-C exerts antioxidant and anti-inflammatory effects, protecting against LDL oxidation and maintaining vascular homeostasis; its reduction is associated with dyslipidemia and diminished insulin sensitivity (51, 52). Measuring UA or HDL-C in isolation may yield inconsistent results due to their functional antagonism and confounding by metabolic factors (17, 31, 53). The UHR, by integrating both molecules into a single metric, more comprehensively reflects the net pro-oxidative burden. An elevated UHR may thus promote stroke through a triad of interconnected pathways: exacerbating endothelial activation, amplifying oxidative stress and inflammation, and reflecting underlying metabolic dysfunction linked to obesity, insulin resistance, and non-alcoholic fatty liver disease (39, 54–56).

Although the model established in the NHANES dataset demonstrated good performance, some discrepancies in the strength of the correlation were observed upon external validation in the Chinese (Shaoyang) dataset. These variations can be plausibly explained by several factors. Firstly, significant heterogeneity exists between the two populations in terms of ethnicity, geographic location, lifestyle, and clinical profiles (e.g., the Shaoyang dataset exhibited a higher prevalence of hypertension and an older mean age). Secondly, differences in healthcare systems and diagnostic criteria may have introduced variations in data measurement and case ascertainment. Crucially, despite differences in magnitude, a consistent positive correlation between UHR and stroke risk was maintained across both datasets, reinforcing the robustness of UHR as a potential biomarker. Our findings emphasize that models derived from specific populations require careful consideration of population characteristics and potential calibration when applied to new settings. Future efforts to refine such models should incorporate more diverse cohorts to enhance their generalizability.

The strengths of this study include its large, nationally representative sample from the NHANES database, which enhances the generalizability of findings. Furthermore, we externally validated the primary outcome model, with comparable results. Secondly, given that both HDL-C concentration and UA are routine indicators that are easy to obtain, they may have considerable potential in the diagnosis and prognosis assessment of stroke.

Several limitations must be acknowledged. First, the cross-sectional design precludes causal inference; the observed correlation requires validation in prospective studies. Second, the occurrence of stroke is a complex process influenced by numerous known and unknown risk factors. Although we adjusted for major known confounders residual confounding from unmeasured or imperfectly measured variables (e.g., dietary habits, physical activity levels, genetic predisposition, or other vascular risk factors) cannot be entirely ruled out. Third, the use of self-reported data for some variables may introduce measurement error. In conclusion, the UHR demonstrates considerable promise as an accessible biomarker for predicting stroke risk, with potential value for risk stratification. However, its generalizability and validity require further verification in large-scale, prospective, multicenter cohorts.

In conclusion, UHR is a readily available and cost-effective biomarker that demonstrates a consistent, positive correlation with stroke risk across diverse populations. Its value lies in its ability to integrate pro-oxidant and antioxidant pathways into a single, clinically accessible measure. Future research should focus on establishing prognostic value in prospective cohorts, defining optimal risk-stratification cut-offs, and investigating whether interventions targeting UHR reduction (e.g., through urate-lowering therapies or lifestyle modifications to improve HDL-C function) can lower stroke incidence.

## 5 Conclusion

This study provides robust evidence from two independent populations that a higher UHR is independently and positively associated with stroke risk. Our findings significantly strengthen and extend prior evidence by validating this correlation in two ethnically and clinically distinct settings—a nationally representative U.S. sample and an independent Chinese dataset—and further by characterizing a linear dose-response relationship. The consistency of the correlation across key subgroups underscores its potential broad applicability.

## Data Availability

The raw data supporting the conclusions of this article will be made available by the authors, without undue reservation.
