# Supplementary material for: The correlation between the uric acid to high-density lipoprotein cholesterol ratio and stroke
Source: Front Med (Lausanne). 2026 Jan 13;12:1720646. doi: 10.3389/fmed.2025.1720646 (PMC12835321; doi:10.3389/fmed.2025.1720646)
Supplement: Supplementary file 1 [file Table_1.DOC]

**Supplementary Table 1** Association between UHR and Stroke in Male(NHANES)

| **Character** | **Model 1** | | **Model 2** | | **Model 3** | |
| --- | --- | --- | --- | --- | --- | --- |
|  | **OR (95% CI)** | **P value** | **OR (95% CI)** | **P value** | **OR (95% CI)** | **P value** |
| UHR | 1.03(1.01, 1.05) | 0.001 | 1.05(1.02, 1.07) | <0.001 | 1.03(1.00, 1.05) | 0.026 |
| **UHR (Quartile)** |  |  |  |  |  |  |
| Q1 | Reference | Reference | Reference | Reference | Reference | Reference |
| Q2 | 0.88(0.64, 1.20) | 0.400 | 0.98(0.71, 1.34) | 0.900 | 0.96(0.67, 1.38) | 0.800 |
| Q3 | 1.49(1.12, 1.98) | 0.007 | 1.68(1.26, 2.23) | <0.001 | 1.51(1.08, 2.12) | 0.017 |
| Q4 | 1.36(0.97, 1.91) | 0.077 | 1.66(1.17, 2.35) | 0.005 | 1.34(0.88, 2.04) | 0.200 |
| **P for trend** |  | <0.001 |  | <0.001 |  | 0.006 |

**Notes:**

- **Model 1:** no covariates were adjusted.
- **Model 2:** age and race were adjusted.
- **Model 3:** age,race,BMI,smoking,drinking,diabetes, hypertension and CHD were adjusted.

UHR is categorized into quartiles (Q1-Q4).
