# Supplementary material for: The correlation between the uric acid to high-density lipoprotein cholesterol ratio and stroke
Source: Front Med (Lausanne). 2026 Jan 13;12:1720646. doi: 10.3389/fmed.2025.1720646 (PMC12835321; doi:10.3389/fmed.2025.1720646)
Supplement: Supplementary file 2 [file Table_2.DOC]

**Supplementary Table 2** Association between UHR and Stroke in Female(NHANES)

| **Character** | **Model 1** | | **Model 2** | | **Model 3** | |
| --- | --- | --- | --- | --- | --- | --- |
|  | **OR (95% CI)** | **P value** | **OR (95% CI)** | **P value** | **OR (95% CI)** | **P value** |
| UHR | 1.09(1.06, 1.11) | <0.001 | 1.07(1.04, 1.09) | <0.001 | 1.03(1.00, 1.06) | 0.044 |
| **UHR (Quartile)** |  |  |  |  |  |  |
| Q1 | Reference | Reference | Reference | Reference | Reference | Reference |
| Q2 | 1.59(1.09, 2.30) | 0.016 | 1.63(1.10, 2.39) | 0.015 | 1.50(1.04, 2.18) | 0.032 |
| Q3 | 1.96(1.35, 2.83) | <0.001 | 1.85(1.27, 2.70) | 0.002 | 1.58(1.08, 2.32) | 0.020 |
| Q4 | 2.62(1.89, 3.64) | <0.001 | 2.24(1.60, 3.13) | <0.001 | 1.53(1.06, 2.21) | 0.024 |
| **P for trend** |  | <0.001 |  | <0.001 |  | 0.070 |

**Notes:**

- **Model 1:** no covariates were adjusted.
- **Model 2:** age and race were adjusted.
- **Model 3:** age,race,BMI,smoking,drinking, diabetes, hypertension and CHD were adjusted.

UHR is categorized into quartiles (Q1-Q4).
