# Supplementary material for: The correlation between the uric acid to high-density lipoprotein cholesterol ratio and stroke
Source: Front Med (Lausanne). 2026 Jan 13;12:1720646. doi: 10.3389/fmed.2025.1720646 (PMC12835321; doi:10.3389/fmed.2025.1720646)
Supplement: Supplementary file 3 [file Table_3.DOC]

**Supplementary Table 3 Association between UHR and Stroke in Male (Shaoyang area)**

| **Character** | **Model 1** | | **Model 2** | | **Model 3** | |
| --- | --- | --- | --- | --- | --- | --- |
|  | **OR (95% CI)** | **P value** | **OR (95% CI)** | **P value** | **OR (95% CI)** | **P value** |
| UHR | 1.05(1.02, 1.09) | <0.001 | 1.05(1.02, 1.09) | 0.004 | 1.04(1.00, 1.09) | 0.082 |
| **UHR (Quartile)** |  |  |  |  |  |  |
| Q1 | Reference | Reference | Reference | Reference | Reference | Reference |
| Q2 | 1.18(0.79, 1.75) | 0.400 | 1.41(0.90, 2.23) | 0.130 | 1.14(0.70, 1.85) | 0.600 |
| Q3 | 1.20(0.81, 1.79) | 0.400 | 1.33(0.84, 2.10) | 0.200 | 1.04(0.62, 1.74) | 0.900 |
| Q4 | 1.41(0.95, 2.10) | 0.088 | 1.37(0.87, 2.16) | 0.200 | 0.97(0.55, 1.71) | >0.900 |

Abbreviations: CI = Confidence Interval, OR = Odds Ratio

**Model 1** : no covariates were adjusted

**Model 2** : adjusted for age, careers, Smoking, Drinking, Hepertension, Diabetes, CHD, Liver disease, kindey disease, and Tumor

**Model 3** : adjusted for age, careers, Smoking, Drinking, Hepertension, Diabetes, CHD, Liver disease, kindey disease, Tumor, SBP, DBP, BMI, TC, TG, HDL-C, and LDL-C
