# Supplementary material for: The correlation between the uric acid to high-density lipoprotein cholesterol ratio and stroke
Source: Front Med (Lausanne). 2026 Jan 13;12:1720646. doi: 10.3389/fmed.2025.1720646 (PMC12835321; doi:10.3389/fmed.2025.1720646)
Supplement: Supplementary file 4 [file Table_4.DOC]

**Supplementary Table 4 Association between UHR and Stroke in Female (Shaoyang area)**

| **Character** | **Model 1** | | **Model 2** | | **Model 3** | |
| --- | --- | --- | --- | --- | --- | --- |
|  | **OR (95% CI)** | **P value** | **OR (95% CI)** | **P value** | **OR (95% CI)** | **P value** |
| UHR | 1.13(1.08, 1.19) | <0.001 | 1.12(1.06, 1.18) | <0.001 | 1.09(1.01, 1.18) | 0.020 |
| **UHR (Quartile)** |  |  |  |  |  |  |
| Q1 | Reference | Reference | Reference | Reference | Reference | Reference |
| Q2 | 3,38(1.91, 6.19) | <0.001 | 2.51(1.35, 4.80) | 0.004 | 1.94(1.00, 3.85) | 0.052 |
| Q3 | 2.39(1.33, 4.44) | 0.004 | 1.96(1.04, 3.78) | 0.040 | 1.29(0.62, 2.72) | 0.500 |
| Q4 | 4.54(2.60, 8.25) | <0.001 | 3.41(1.84, 6.51) | <0.001 | 2.32(1.08, 5.11) | 0.034 |

Abbreviations: CI = Confidence Interval, OR = Odds Ratio

**Model 1** : no covariates were adjusted

**Model 2** : adjusted for age, careers, Smoking, Drinking, Hepertension, Diabetes, CHD, Liver disease, kindey disease, and Tumor

**Model 3** : adjusted for age, careers, Smoking, Drinking, Hepertension, Diabetes, CHD, Liver disease, kindey disease, Tumor, SBP, DBP, BMI, TC, TG, HDL-C, and LDL-C
